# Therapeutic nanoparticles penetrate leaves and deliver nutrients to agricultural crops

**DOI:** 10.1038/s41598-018-25197-y

**Published:** 2018-05-17

**Authors:** Avishai Karny, Assaf Zinger, Ashima Kajal, Janna Shainsky-Roitman, Avi Schroeder

**Affiliations:** Department of Chemical Engineering, Technion – Israel Institute of Technology, Haifa, 32000 Israel

## Abstract

As the world population grows, there is a need for efficient agricultural technologies to provide global food requirements and reduce environmental toll. In medicine, nanoscale drug delivery systems grant improved therapeutic precision by overcoming biological barriers and enhancing drug targeting to diseased tissues. Here, we loaded nanoscale drug-delivery systems with agricultural nutrients, and applied them to the leaves of tomato plants. We show that the nanoparticles – liposomes composed of plant-derived lipids, penetrate the leaf and translocate in a bidirectional manner, distributing to other leaves and to the roots. The liposomes were then internalized by the plant cells, where they released their active ingredient. Up to 33% of the applied nanoparticles penetrated the leaf, compared to less than one percent of free-molecules applied in a similar manner. In our study, tomato plants treated with liposomes loaded with Fe and Mg overcame acute nutrient deficiency which was not treatable using ordinary agricultural nutrients. Furthermore, to address regulatory concerns regarding airborne nanoparticles, we rationally designed liposomes that were stable only over short spraying distances (less than 2 meters), while the liposomes disintegrated into safe molecular building blocks (phospholipids) over longer airborne distances. These findings support expanding the implementation of nanotechnology for delivering micronutrients to agricultural crops for increasing yield.

## Introduction

Improving the uptake of active agricultural ingredients is necessary for minimizing biotic stress and enhancing yield^1,2^. In fact, it is assumed that only 0.1% of applied crop-protection-agents reach their biological target, whereas the majority are lost to the environment^3^.

To reach their target, Agricultural ingredients are applied to crops either by irrigation or directly to the foliage. Foliar feeding circumvents problems associated with soil penetration and biodegradation. The efficiency of foliar applications strongly depends on absorption and mobility, where in many cases, both requirements are lacking^4–6^.

For foliar feeding to be effective, agricultural ingredients must penetrate the leaf’s barriers. There, the agricultural ingredients can either reside locally within mesophyll tissue or be transported to other plant regions through vascular bundles. Translocation beyond the leaf is mediated by apoplastic (xylem) and/or symplastic (phloem) pathways^7^; the former corresponds to movement around cell walls and intercellular spaces, and the latter is defined by the translocation from one cell to another through the plasmodesmata, i.e. cell-to-cell cytoplasmic linkage. Once an agricultural ingredient enters the cell, it usually targets an organelle, typically the chloroplast, or affects metabolic processes in the cytoplasm.

Various technologies are available for delivering agricultural ingredients to seeds or to cells in culture, such as electroporation and ultrasound^8,9^. However, modalities designed to improve delivery to the whole plant are necessary to address gaps in agricultural research and practice. Nano-carriers hold great promise for bridging this gap, due to their ability to carry complex payloads across biological barriers and target to specific tissues^10–12^. Specifically, we sought to test medical nanoparticles, composed of biocompatible and biodegradable lipids, for agricultural use.

Liposomes, vesicles with an inner aqueous core surrounded by a lipid bilayer, are widely used as carriers of medicinal small molecules, proteins and nucleic acids^13,14^. Liposomes are stable in aqueous environments and subsequently fuse to the plasma membrane or are internalized by cells through endocytic and signaling pathways^15^. The release kinetics of active agents from the liposome can be tailored by modulating the physicochemical properties of the lipid bilayer, namely by altering the phase transition temperature (Tm) of the liposomal membrane or by enriching the bilayer with sterols, such as cholesterol^16^. External stimuli, such as light waves, heat and pH, have been used as triggers for controling the release of payloads of liposomal payload release spatially and temporaly^17^. Polyethylene-glycol (PEG) and targeting moieties conjugated to the surface of the liposomes have been shown to improve targeting to specific tissues^18,19^.

**Figure 1:**
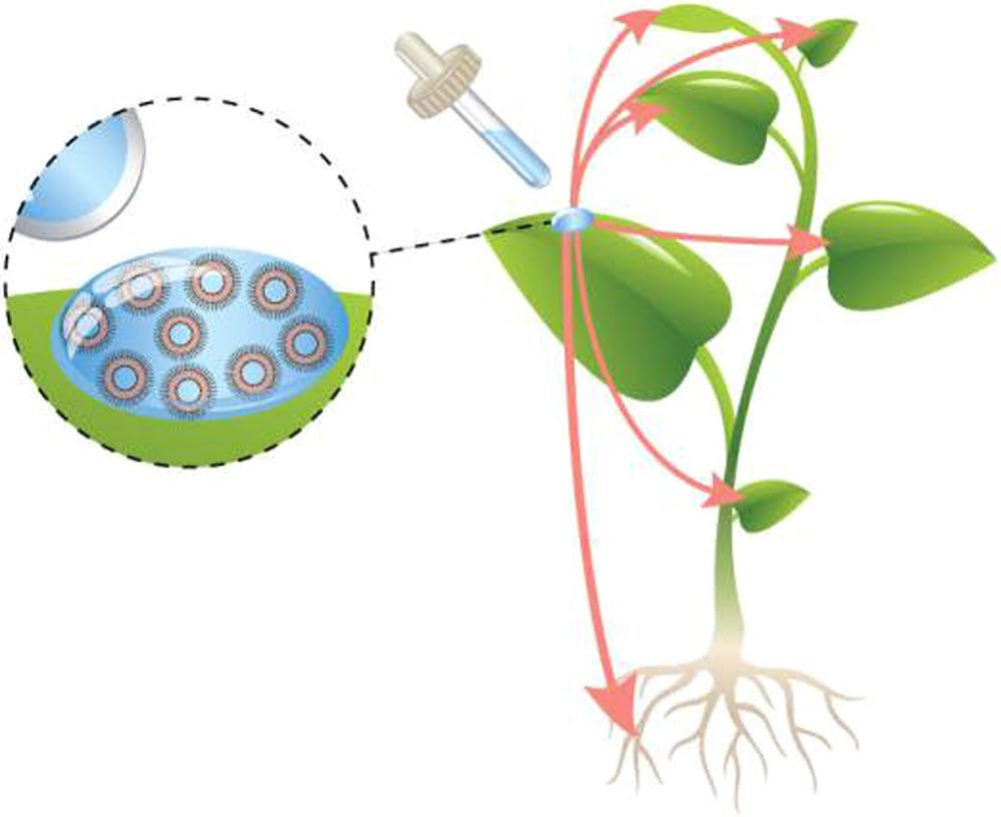
Study overview. One-hundred nanometer liposomes were evaluated for their capacity to deliver active agricultural ingredients to fully grown crops, after being applied foliarly. The liposomes penetrated the leaves and translocated to the entire body of the plant - roots and leaves, releasing their payload inside the cells. This approach was then used to supplement plant growth and overcome acute nutrient deficiency.

In this study we sought to adapt medical liposomes for delivering agricultural ingredients to plants. We tested the ability of 100-nm liposomes to deliver agricultural nutrients to seedlings and to fully-grown tomato plants, examining the biodistribution and activity of the nanoparticles from a single leaflet to the entire plant (Fig. 1).

## Results and Discussion

A favorable route for administrating active ingredients to plants is directly to the foliage; however many agricultural ingredients cannot be administered in this manner due to poor leaf penetration and intra-plant mobility^20^. Nanoparticles have proven to be effective carriers of a wide scope of compounds, capable of enhancing biological targeting, delivery and uptake^17,21,22^. Here, we tested medical nanoparticles, specifically – 100 nm PEGylated liposomes, for their potential agricultural use, as carriers of agricultural ingredients to plants.

### Nanoparticles (NPs)

Liposomes are vesicles comprised of phospholipids that form a bilayer membrane that surrounds an inner aqueous core^23^. We chose to construct the liposomes from soy-derived phospholipids, in order to reduce hypersensitive responses plants may have to foreign, non-plant, materials^24^. Specifically, the liposomal formulation was based on a 16/18C-chain lecithin (hydrogenated soybean L-α-phosphatidylcholine, HSPC). HSPC is also the primary phospholipid in the clinically-approved anti-cancer liposomal drug Doxil^25^.

From a biophysical perspective, the saturated 16C and 18C acyl tails on the phospholipids grant a high gel-to-crystalline phase transition temperature (T_m_ = 52.5 °C), reducing heat- and osmotically-mediated destabilization of the liposomal membrane in agricultural conditions. We tested the foliar uptake of particles with a diameter of 100 nanometers, which have been shown to be stable for long periods of time and effective in penetrating biological systems for medical applications^26^.

### Nanoparticles translocate from the leaves to the roots and to adjacent leaves, after foliar application

**Figure 2:**
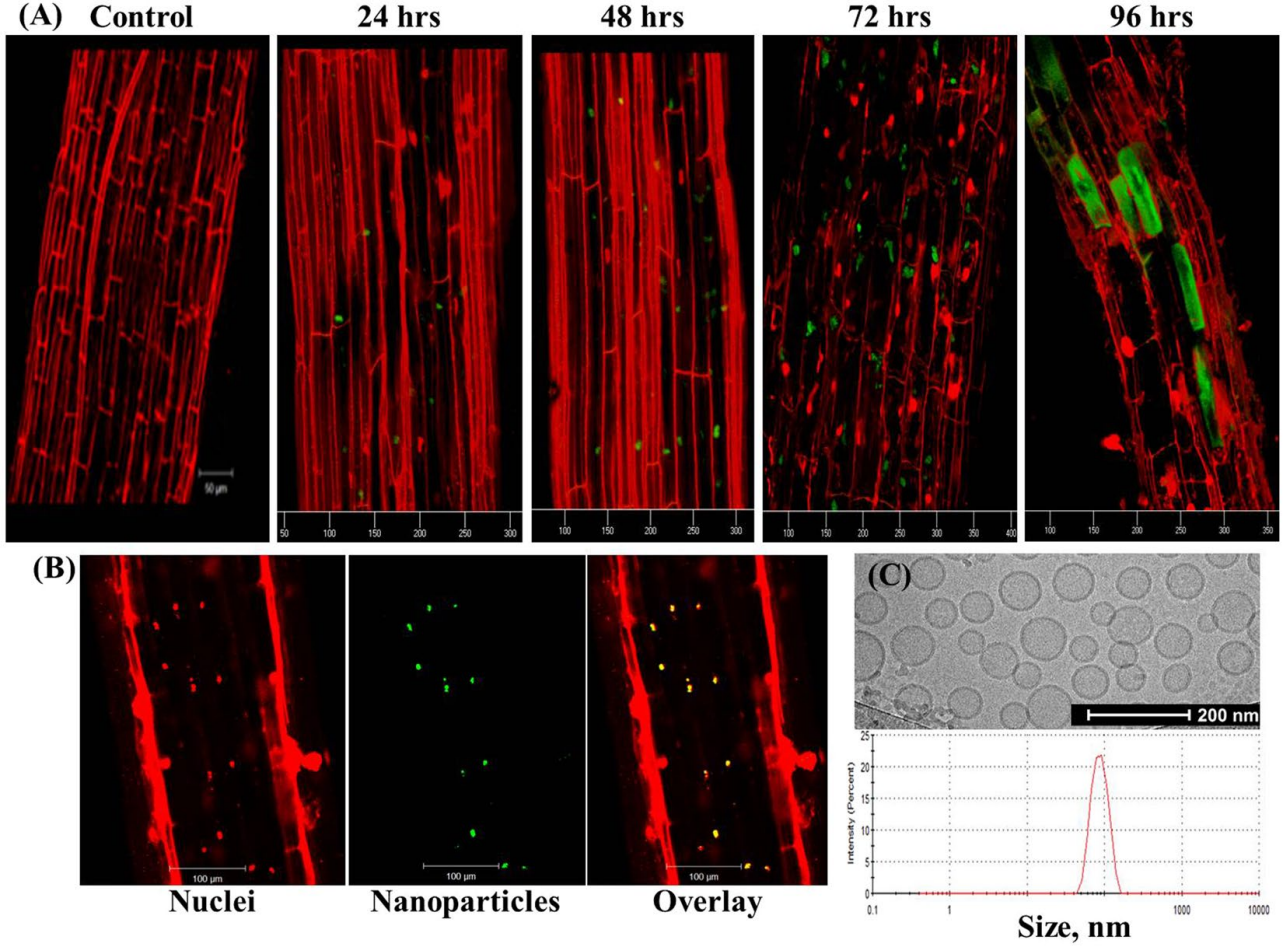
Nanoparticles applied to the leaves translocate to the plant roots. **(A)** Liposomes containing a fluorescent tracer (fluorescein, green) were applied foliarly to cherry tomato plants. Secondary and tertiary roots were imaged over a period of 96 hours, using confocal microscopy (the plant plasma membrane is stained red). During the first 72 hours post application, particles gradually accumulated in individual root cells. 96 hours post foliar application the liposomes disintegrate and release their cargo into cytoplasm. *Lower panel* – confocal images of a tertiary root 72 hours post foliar application. **B**_left_ - propodeum iodide (PI) stained root nuclei (red) and the corresponding fluorescent nanoparticle distribution (green, B_middle_), as well as the overlay of both imaging channels (**B**_right_), indicating the nuclei colocalization of the particles. (**C**) Particle size was measured using dynamic light scattering and cryoTEM, having a mean particle size of 88.37 ± 21.13 nm (PDI = 0.037).

Liposomes, 100-nm in diameter, loaded with fluorescein, were applied to a single apical leaflet of cherry-tomato plants (*Solanum Lycopersicum var. cerasiforme*). Primary, secondary and tertiary roots were washed, segmented and imaged using confocal microscopy. Time-dependent accumulation of the liposomes was observed in the roots already 24 hours post foliar application, peaking at 72 hours (Fig. 2A). Intracellularly, the liposomes were found to be closely associated with the nuclei (Fig. 2B), where they gradually formed aggregates. 96-hours post application the entire cell body was stained fluorescently (Fig. 2A, 96 hr), suggesting release of the dye from the liposomes. Intracellular cargo release can be mediated by the disruption of the nanoparticle by lipases, or due to dye leakage caused by osmotic destabilization^27,28^.

**Figure 3:**
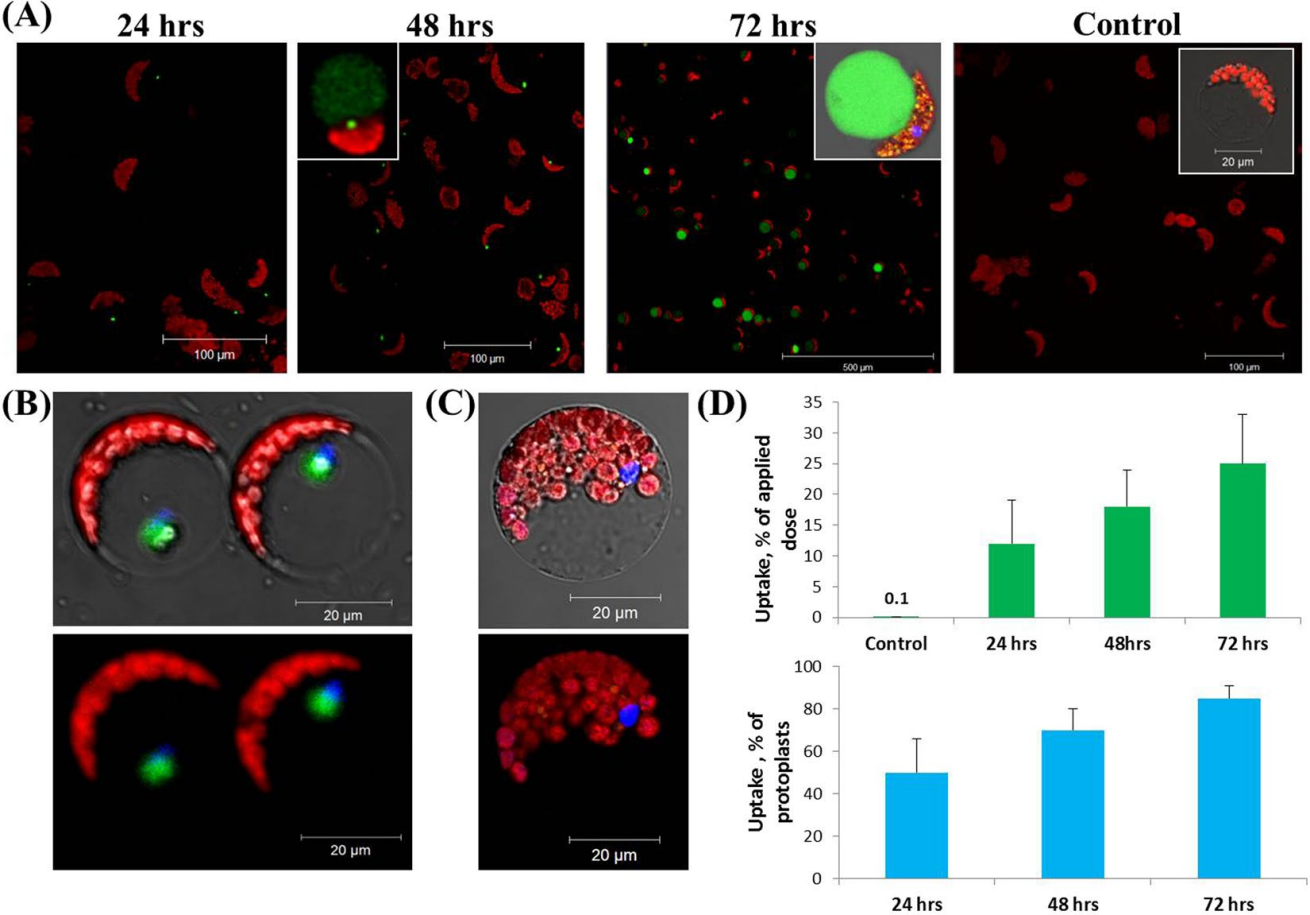
Nanoparticles applied to a single leaf translocate to adjacent leaves. After applying the nanoparticles to a single leaflet, the nanoparticles were found also in the adjacent leaves, above and below the application site. Isolated mesophyll protoplasts from adjacent leaflets were observed 24–72 hours post foliar application using confocal microscopy. *Upper panel* (A) 24 hours post application, particles (green) are detected within cells; 48 hours post application the fluorescent signal from the cytoplasm increases, notably seen in upper-left insert. 72 hours post application a strong fluorescent signal is detected from the cytoplasm and vacuole, concomitant with release of internal cargo. Control – Protoplasts isolated from non-treated plants exhibit a minimal fluorescence signal compared to the treated cells (upper sub-image). (**B**) Co-staining of the leaf cell nuclei (blue) and the fluorescent liposomes (green) 24 hours post foliar application (*upper* – bright field and fluorescence overlay, *bottom* – fluorescent). The fluorescent liposome signal appears closely associated with the nucleus, compared to (**C**) nuclei-stained protoplasts isolated from non-treated controls. (**D**) The % of cells visualized with nanoparticles inside them, and the overall % of administered liposome dose that was taken up by the plant. (Upper graph) Uptake rate – the % of nanoparticle that were absorbed by a single leaflet relative to the amount applied, ranged 20–33%. (Lower graph) the amount of cells detected with nanoparticles inside them after the treatment reached 70–90%, 72 hr post foliar application.

We then tested the distribution of foliarly-administered liposomes to adjacent leaves. Liposomes (100 nm, Fig. 2C) were applied to a single apical leaflet. Twenty-four hours later, adjacent leaflets were stripped from the epidermal tissue and digested enzymatically to obtain mesophyll protoplasts (Fig. 3A–C). Liposomes were detected in 50 ± 12% of protoplasts 24 hours post application, and in 85 ± 7% of protoplasts after 72 hours (Fig. 3D, lower panel). In all cases the liposomal fluorescent dye was disassociated from the chloroplast, yet closely associated with the nucleus (Figs 2B and 3B). These observations indicate that liposomes translocate through the leaf to other leaves and roots, subsequently releasing the cargo in the nucleus or cytoplasm.

### Quantitating the biodistribution of nanoparticles post foliar application

**Figure 4:**
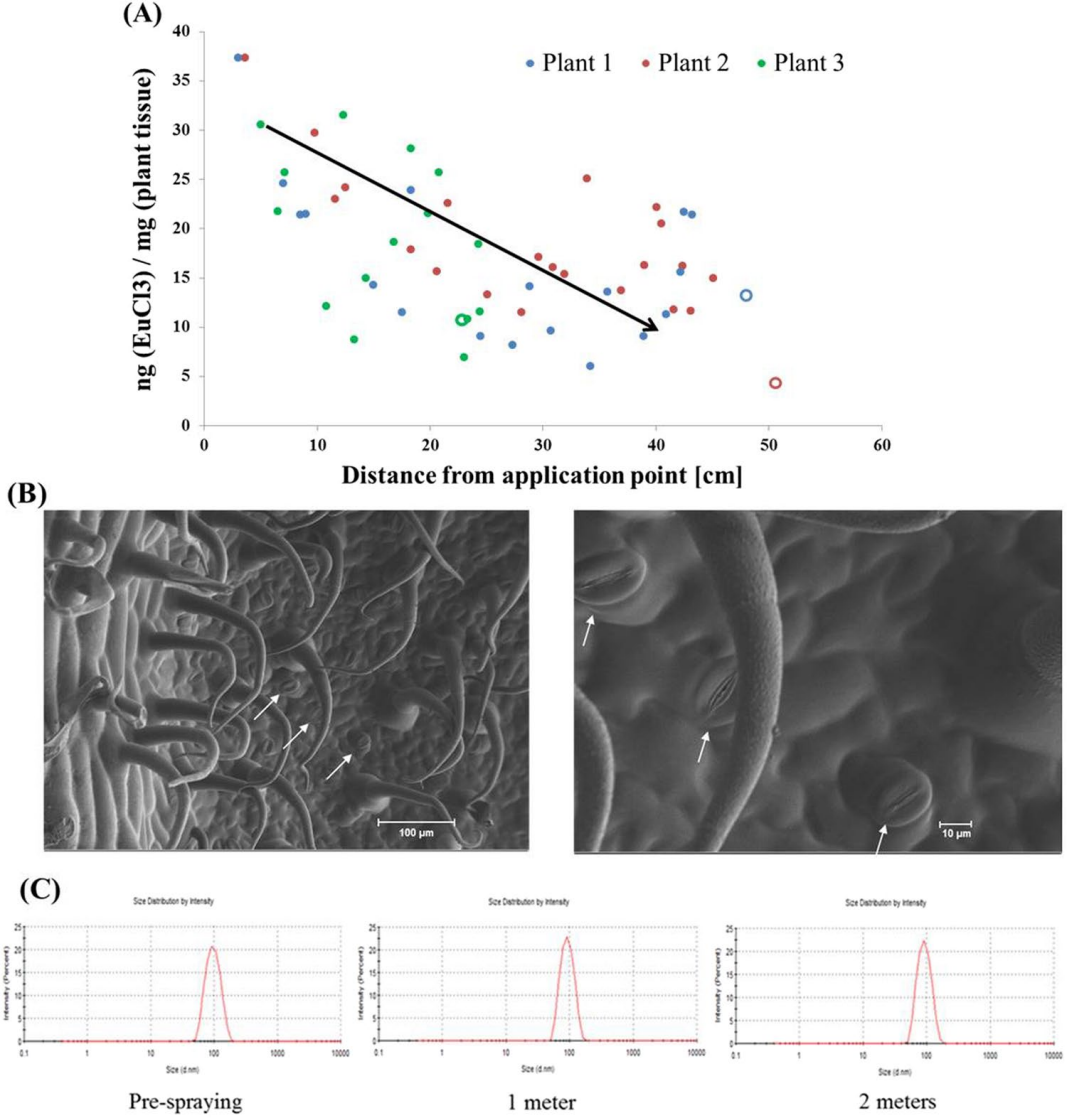
Nanoparticle concertation decreases as the distance from foliar application site increases. Liposomes loaded with the rare earth Eu were applied to a single leaflet of tomato plants. The Eu content was measured in multiple plants as a function of the distance from the application site. Three representative plants are displayed. As the distance from the application site increases, the Eu concertation decreases. The hollow data points correspond with each plant’s roots. (**B**) Scanning electron microscopy (SEM) images showing the adaxial surface of tomato leaves (**A**) hairy-like cuticles cover the leaf surface, and (**B**) stomata. Images feature trichomes (leaf hairs) and leaf stomata. White arrows mark for focused stomata, with pore size ranging 8–20 µm, distributed every 30–50 µm. *Vertical drift and stability of liposomal formulation in spray.* 100-nm liposomes were charged into a commercial agricultural spraying device and sprayed over distances of 1 and 2 meters (n = 3). Liposomes retrieved from plate were sized. Poly Dispersity Indexes (PDIs) were of satisfactory. *Long distance spray*. 100 nm liposomes were sprayed through a fan-facilitated wind tunnel in horizontal direction to a distance of 8 meters, and later collected from a awaiting corrugated plastic screen: Particles were undetectable.

The ability of liposomes to deliver sufficient amounts of agricultural ingredients to plants was tested. Liposomes were loaded with a tracer molecule – the rare earth europium (Eu), and applied to a single upper leaflet in each plant. 24, 48 and 72 hours later, all the plant’s leaflets were sectioned from their petioles, digested with acid, and analyzed for Eu content *via* elemental analysis relative to their distance from the application point (Fig. 3D, upper panel, Fig. 4).

Foliarly-applied liposomes were detected in declining molar concentrations as the distance from application point increased (Fig. 4A). However, all treatment measurements had an Eu content above 100 ppb, which is greater than the amount necessary for treating micronutrient deficiencies^29^. Furthermore, 24 hours post application the measured uptake ranged 7–19% of the foliarly-applied dose and after 72 hours it reached 27–33%. When Eu was applied in a free molecular form (without a nanoparticle), the uptake was below 0.1% (Fig. 4A). Together, these data demonstrate that nanoparticles grant an advantage in penetrating the leaf and delivering sufficient amounts of agricultural ingredients to the plant tissue. While we were unable to definitively determine the route through which the liposomes penetrate the leaves, one possibility is that the nanoparticles penetrate through the stomata, which have pore sizes ranging from 8 to 20 µm (Fig. 4B). Scanning electron microscopy (SEM) images of the adaxial surface of tomato leaves demonstrated the trichomes (leaf hairs) covering the leaf surface and the close proximity to the stomata.

### Short-range, but not long-range, nanoparticle stability in spray

There is growing regulatory concern regarding the drift and inhalation of airborne nanoparticles^30–32^. To address this, we engineered nanoparticles that are stable over short distances of agricultural spraying (<2 meters), but disintegrate into biocompatible phospholipids molecular building blocks over longer distances. 100-nm liposomes were sprayed and collected over different distances. At short distances, i.e., 1 and 2 meters, we were able to collect the liposomes on a capture plate (Fig. 4C). The particles collected in the capture plates after 1 and 2 meters had a single size population and deviated only by 5% from the initial population of pre-sprayed liposomes. However, over longer distances (8 meters), using a wind tunnel to simulate drift, we were unable to detect the nanoparticles. This can be explained by inability of the liposomes to remain intact in a dry environment, as lipid self-assembly is dependent on an external hydrating environment^33^.

### Supplementing nutrient deficiency in tomato plants

**Figure 5:**
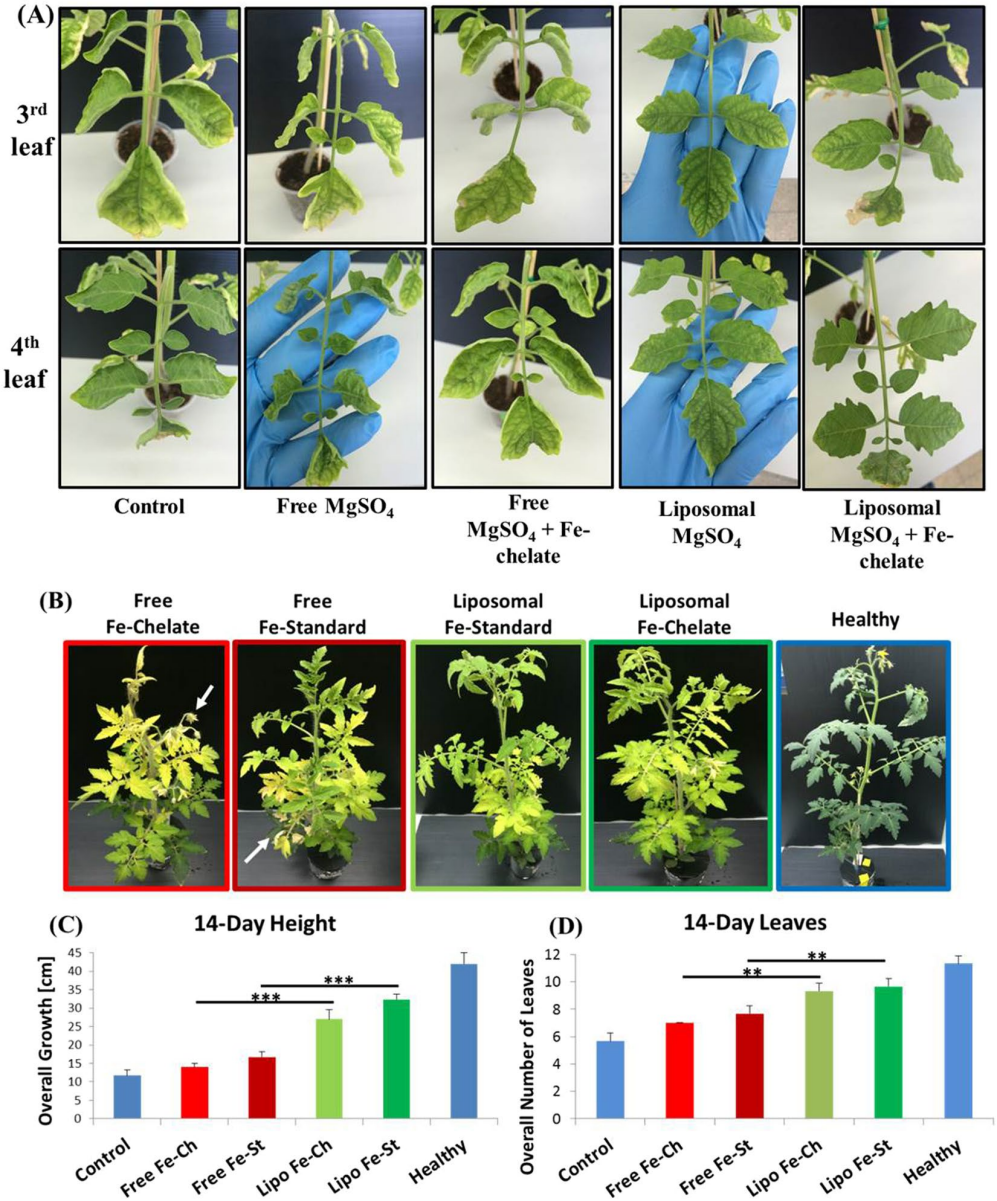
Using nanotechnology to supplement Mg and Fe deficiencies in tomato plants (Upper panel). Tomato seedlings were grown in micronutrient-poor soil for 3–4 weeks until having 5 fully emerged leaves (Supp. 5). Afterwards, a single apical leaflet of the 5^th^ leaf in each plant was sprayed with 20 µl of the indicated treatment. Plants were monitored for changes in leaf coloring, structure, and overall growth rate. Images taken on day 14 show 3^rd^ and 4^th^ leaves improved recovery in the plants treated with the liposomal formulation, versus plants treated with same amounts of non-encapsulated formulations. *Treating Fe-deficiency over a hydroponic system (lower panel).* Tomato plants were grown in a nursery. After being stripped from the soil and adjusted to hydroponic growth system (Supp. 6), they were transferred to a Fe-deficient Hoagland media. The apical leaflet in each plant was treated according to the panel (30 µl). 14 days post the 1^st^ application, plants showed distinctive differences in growth and recovery. While healthy control specimens appear lush green, plants treated foliarly with a commercially available non-liposomal Fe-chelate displayed complete yellowing and necrosis of upper and new growth (red arrows). Similarly, free Fe-standard shows acute yellowing and moderate necrosis of lower leaves. Liposomal Fe-chelate shows moderate yellowing of leaves w/o necrosis and light-green new growth, and liposomal Fe-standard shows moderate yellowing of upper leaves, no necrosis and light-green new growth. Growth patterns were altered as free-form treated plants essentially stopped promoting new growth, whereas both forms of the nanoparticle liposomally-encapsulated iron promoted new healthy growth reaching 75% of healthy plants that didn’t undergo any stress. ***P < 0.001, **P < 0.05 in a standard student t-test.

Tomato plants with Mg and Fe deficiencies were sprayed with a commercial Mg solution and iron, or with 100-nm liposomes loaded with Mg or Fe. Specifically, a single apical leaflet was administered 20 µl of the liposomes or of the corresponding non-encapsulated commercial supplement. Recovery was examined in the leaves adjacent to the application site. 14 days post application, significantly improved recovery (P < 0.01) was recorded in the groups of the liposomal treatments compared to commercial formulations (Fig. 5A–D). The free (non-encapsulated) nutrient application did not restore chlorosis or epinasty, and mild necrosis began along the 3^rd^ leaf curled-edged leaflets (Fig. 5A). Contrarily, liposomal formulations restored both chlorosis and epinasty, with strengthening of turgor pressure in the leaves. Effects were notably seen among the emergence of secondary symptom-free leaflets, implicating the recovery from the nutritional distress and activation of plant-growth mechanisms.

To further address a pre-determined nutritional deficit in the plant, we imposed a single-element (Fe) deficiency using a Hoagland hydroponic growth media. Foliarly-applied Fe liposomal supplements were successful in restoring plant vitality and reducing necrosis. Even in comparison to fully nourished controls, foliarly-treated plants reached 75% of the healthy growth size (Fig. 5). In contrast, plants treated foliarly with the free nutrient failed to continue growing and became necrotic. These data suggest that using a protective nanocarrier ensured intra-cellular translocation of nutrients without the need of using chemical chelators to protect the Fe. Overall, these results show that nanotechnology grants a new technological alternative for treating fully-grown crops.

### Adapting medical liposomes to agriculture

Since first described in the late 1960s liposome-based technologies have translated into numerous pharmaceutical practices^34^. Recent reductions in lipid cost have opened the opportunity to expand the technology to the agricultural sector, where costs-per-acre must be kept affordable.

Here, we demonstrated the capacity of 100-nm soy-based liposomes, through single-leaflet application, to penetrate and distribute to the whole plant. Distribution and payload delivery was examined both qualitatively and quantitatively.

### Nano-liposomes translocated through plant tissues

Time-dependent accumulation in the cytoplasm and nuclei of both root cells and photosynthetic tissues in adjacent leaves was observed, followed by compartmentalization of the released cargo into cells vacuoles. A 72-hour uptake mark was measured by quantifying the liposome’ biodistribution within the plant biomass. Higher uptake levels were found using this method, compared to molecular application, and efficacy was demonstrated in supplementing plant nutritional deficiencies. We found that compounds applied foliarly in a nanoparticulate form had above 30% uptake, far superior to the 0.1% uptake previously reported^3^.

To determine the efficacy in supplementing nutritional deficiencies, two growth scenarios were examined. Firstly, we treated plants grown in soil and diagnosed with micronutrient deficiencies. Secondly, a hydroponic Hoagland setup was used to impose a deficiency, supplemented by foliarly applied liposomal treatment over a single leaflet. Both experiments showed enhanced productivity of liposomal-encapsulated elements over its free-form correspondent and the insignificance of chelate agents to protect the metal as mediators.

These findings demonstrate that nano-liposomes penetrate foliage and translocate bi-directionally throughout the plant, taking advantage of the plant’s natural transportation mechanisms and without exerting any toxicity. Liposomes were seen closely associated with cells nuclei and released their cargo after more than 48 hours, where liposomal membrane disruption seems to be as a result of endogenous factors (e.g. cytoplasmic lipases) and osmotic destabilization. Expanding the use of nanotechnology to enable the delivery of active compounds to plants holds promise to improve agricultural research methodologies and increase yields.

## Materials and Methods

Liposomes averagely sized 95 ± 25 nm were loaded with fluorescent dyes, rare earths or nutritional agents. The biodistribution of nanoparticles (NPs) and subsequent release rates of the compounds at the biological site were demonstrated on Cherry Tomato (*Solanum Lycopersicum var. cerasiforme*) by both qualitative and quantitative approaches. Samples were taken from different parts of the plant at predetermined time intervals post application. Visualization of biodistribution and capacity to penetrate the cell wall was performed using Confocal Laser Scanning Microscopy (Olympus LSM 510 Meta, Tokyo, Japan). Quantification of uptake extent was determined by Inductively-Coupled-Plasma Optical-Emission-Spectrometry (ICP-OES, Agilent 5100VDV, Santa-Clara, USA).

### Liposome synthesis, encapsulation and characterization

The liposomal formulation was produced using solvent-injection technique^35^, and modified to a 50 mM formulation of HSPC (L-α-phosphatidylcholine hydrogenated from soy bean), cholesterol and PEG-DSPE 2000 (1,2-distearoyl-*sn*-glycero-3-phosphoethanolamine-N-[amino(polyethylene glycol)-2000] (ammonium salt)) (Avanti Polar Lipids, AL, USA). The lipid mole ratio being 30:19:1 respectively, and solvent being 5% D+−Glucose (DEX, Sigma Aldrich, MO, USA) in double-distilled water (DDW). HSPC (hydrogenated soy phosphatidylcholine) was chosen as dominant structural lipid for this platform due to being naturally driven from plant tissue and able to overcome the Hypersensitive-response usually associated with the introduction of xenobiotics and potential pathogens to plants^36^.

Preparation was obtained in batches by forming separate lipid and aqueous phases, followed by the injection of the 10% v/v lipid phase into the 90% v/v aqueous phase. Both preheated to 65 °C and slightly vortexed upon mixture to achieve homogeneity. Size and poly-dispersity decrement were attained by Nitrogen-facilitated and poly-carbonate membrane-mediated stepwise extrusion followed by 12–14 kD cut-off cyclic dialysis for dismissal of non-encapsulated residues. Post dialysis, solution was analyzed for size cultures and dispersity through dynamic light-scattering (DLS) (Malvern Instruments, Malvern, UK) in both dilutions of 1:100 and 1:10 as part of ordinary dual-test procedures. Liposomes were either utilized for plant applications or stored at 4 °C for future use. Each batch was resized if used for more than 7 days from its synthesis, where overall stability of stored solutions exceeded the 90 day-mark (not shown).

For the encapsulation of hydrophilic or charged compounds, i.e. passive loading, an organic phase is prepared by dissolving the lipid mixture in EtOH (10% vol. from final volume of organic and inorganic phases combined) at 65 °C. An aqueous phase is prepared separately by dissolving the compound of Interest (COI) in 5% D+−Glucose (Dextrose, DEX) using (DDW) as solvent, at 65 °C. Upon reaching complete homogeneity within organic tube, merging of both solutions is obtained by injecting the organic phase into the inorganic phase in one rapid and consistent motion. Both clear solutions merged to one cloudy solution and achieved homogeneity. The mix of spontaneously-formed encapsulating vesicles is downsized by pure nitrogen-facilitated stepwise extrusion (Northern Lipids 20 ml Lipex extrusion apparatus, circulating hot tub by Fisher Scientific, Inc.). This is done through two 25 mm dual-size polycarbonate membranes, resting on top of an essential drain disk (from Whatman^TM^ Labware, Maidstone, UK), initially introducing 400, 100, 80 and then 50 nm membranes. The extruded solution goes through 12–14 kD cutoff dialysis tubing (Spectrum Laboratories, Inc.) at 4 °C, overall five repetitions each two hours long.

For active loading, a batch of liposomes containing 100 mM calcium acetate (Sigma Aldrich) or ammonium sulfate were prepared by passive loading as described above. Following dialysis, the particle solution is introduced with the COI (dissolved in 5% DEX) at 55 °C for 60 minutes. The osmotic pump generated by salt-encapsulation works in parallel and inversely with the concentration gradient.

Post synthesis characterization included sizing of NPs through Dynamic Light Scattering (DLS, Malvern Zeta-sizer Nano), performed in both dilutions of 1:10 and 1:100 as part of dual-testing procedures, and Entrapment Efficacy (EE) achieved by either Plate reader (Tecan Infinite M200 pro) or ICP-OES.

Cryo-TEM. Cryogenic transmission electron microscopy (cryo-TEM) was used to image the liposomes, as described by Talmon^37^. We used an FEI Talos 200 C Field Emission Gun (FEG) high-resolution TEM. The samples were mounted on Gatan 626 cryo-holders to maintain cryo-preservation in the TEM at −180 °C. Images were recorded using a FEI Falcon III camera, the contrast was enhanced using a Volta Phase Plate.

### Indoor Growth System

Plants were grown separately within an indoor growing chamber (Secret Jardin DS90, Brussels, Belgium) powered by 4 × 65 W white light fluorescent lamps (EuroLux^TM^, total emission ~16 K lumens) designed for plant growth. The plants were anchored symmetrically to a Mylar-coated surface for maximum reflection. Ventilation was kept constant using simultaneous inlet/outlet blowers, in capacity ratio of 3/4 respectively. This facilitated a slightly negative pressure within the chamber, designed for airborne pest-control. Growth substrate was composed of 0–40 mm Kabul (Peat), sterilized coconut fibers, 7% sea-sand quartz, 1.5 Kg/m^3^ 14–14–14 “Osmocot” (from Danor, Gan-Haim, Israel) and 750 gr/m^3^ 24–5–8 starter fertilizer. Seeds were germinated uniformly by being soaked in deionized water for 24 hours, followed by a 72 hour sprouting period inside a moist-cloth-padded, sterilized (Tuttnauer steam sterilizer 3870EL, NY, USA) and paraffin-sealed petri dish. The seeds were grown in growth substrate between 3–6 weeks (end of seedling phase and beginning of vegetative phase, according to necessity) before being subject to NPs application. Monitoring and modification of relative humidity (40–60%), temperature (22–28 °C) and CO_2_ levels (250–450 ppm) was obtained by using Rotronic CX11 3-way gauge (Bassersdorf, Germany). This was carried out in order to ensure uniform and optimal growing conditions as chemical-containing NPs were applied to the canopy, lower leaves or roots.

### Bio-distribution measurements (Qualitative analysis) in roots

Plants were grown from germinated seeds within growth substrate up to 3–5 weeks (5–8 leaves) according to necessity, followed by stripping from growth substrate and transfer to plastic tubes containing tap-water nutrition media (pH = 7.2 ± 0.2).Upon adjustment to hydroponic media and transplant-recovery, an upper apical leaflet was submerged *in-vivo* within 10 ml of 1:2 diluted Fluorescein liposome solution for 96 hour period (Fluorescein – free acid, λ_ex_/λ_em_ = 496/516 nm, Sigma Aldrich, MO, USA). Secondary and tertiary roots were cut from the living root-ball at predetermined time intervals post application; they were fragmented, mounted on 1–1.2 mm thick microscopic slides, added with 50–100 µM of Propidium Iodid (depending on root thickness, λ_ex_/λ_em_ = 560/608 nm) and analyzed via Confocal Laser Scanning Microscopy, where 3D-stacks were obtained and interpreted.

### Bio-distribution measurements (Qualitative analysis) in leaves

Plants were grown according to the described; an upper leaflet was submerged within 10 ml of 1:2 diluted (from bulk) Fluorescein-containing liposomes for 72 hours, and leaflet samples were collected from adjacent leaves every 24 hours. Samples were thoroughly washed with 10% EtOH followed by DDW, and left within pre-prepared tissue-digestive enzyme solution over-night (O.N.) to facilitate protoplast isolation (modified protocol can be seen below). Prior to slide-mounting, additional fluorescent compound was added for nuclear staining (Hoescht 33342, λ_ex_/λ_em_ = 350/453 nm, Molecular Probes^®^, OR, USA) to a final concentration of 0.1 mg/ml.

### Protoplasts isolation from Tomato leaves

Solution is based on the protocol presented by Sang-Dong Yoo *et-al*. 2007, modified for Cherry Tomato (*Solanum lycopersicum var. cerasiforme,* Table 1).

**Table 1:** Preparation of tissue-digestive enzyme solution. A stock of the stated materials should be prepared beforehand and kept in 4 °C if stored.

Group	Component	Prepared stock	Amount added (per 1 ml solution)
Medium	0.4 M Mannitol	—	73 mg
	20 mM KCl	1M	20 µl
	20 mM MES (pH = 5.7)	0.5M	40 µl
Enzymes	2.5–3% Cellulase R10	—	25 mg
	0.66–0.8% Macerozyme R10	—	7 mg
Stability	10 mM CaCl_2_	1M	10 µl
	0.1% BSA (Sigma A-6793)	—	1 mg

All components obtained from Sigma Aldrich, MO, USA.

For batch preparation of digestive enzyme solution, 80% of the final volume was primarily added with DDW. This was followed by the addition of Medium group, immersion within 70 °C-preheated bath for 3–5 minutes and cooling back to room temperature (RT). Next, the enzymes group is added, slightly vortexed to homogeneity and heated to 55 °C for 10 minutes to inactivate proteases and enhance enzyme stability. This is followed again by cooling to RT and adding of stability group. DDW is added to achieve the total volume required, yielding a light-brown colored, clear enzyme solution, passed through 0.45 µm syringe filter.

Preparation of leaf specimen was conducted by washing the leaflet with flowing tap water for 30 seconds, and piercing it (approx. every 1–2 mm) using a sterile surgical blade. This enlarges the exposed surface and cell wall allowing for rapid enzymatic degradation. Finally, the pierced leaflet sample is immersed within 2.5 ml enzyme solution, using 1.5″ diameter petri dish, incubated O.N. in dark conditions at 37 °C w/o shaking.

Digested samples were gently washed with its own matrix using topped-tip pipette, in order to encourage semi-degraded tissue to be washed off from its scaffold. This was mixed with a nuclear staining agent (Hoechst 33342, from Sigma Aldrich) to a final concentration of 0.1 mg/ml, and centrifuged at maximum 500 × g for 5–10 minutes. Protoplast precipitate was collected using topped-tip pipette and loaded into 6-channel µ-slides (VI^0.4^, from Ibidi, Martinsried, Germany) and kept away from direct light before being analyzed by CLSM.

### Bio-distribution measurements (Quantitative analysis)

Plants were grown for 3 weeks and transferred to hydroponic tap-water media where they were left to adjust and proceed growth up to 5–8 weeks (8–12 leaves). Then, we applied apical leaflet-submerging (one leaf below new growth), within rare-earth-metal-encapsulated liposomes for 72 hour period (EuCl_3_, from Sigma Aldrich). Encapsulated material within NPs was ~25 ppm, obtained by proper dilution of bulk liposomes solution. Following exact distance-from-application-point measurement for each future sample, plants were dismembered to their separate leaflet and root samples, followed by 2 hours @ 105 °C oven dehydration (BIFA Electro-therm MS8 multi stage laboratory furnace, max temp. 550 °C, Middlesex, UK) and dry weight measurements. Plant samples were later placed within ceramic bowls and fully digested by 5 hours @ 550 °C cremation. Ash residues were dissolved in 1% Nitric-acid and collected to tubes, where final volume for each sample predetermined to 10 ml. Samples were filtered through 0.45 µm syringe filter (Millipore Millex Syringe-driven filter unit) and analyzed for Europium content via ICP-OES apparatus.

### Hydroponics, imposing deficiency and supplementing foliar applications

Seeds of uniform genetics were germinated inside moist-cloth-padded and Paraffin-sealed petri dishes, and grown in a designated nursery for approx. 3 weeks (~4^th^ leaf). They were then stripped from the soil and washed thoroughly with DDW. Each plant’s exposed root system was immersed in 250 ml of full 0.5 Hoagland solution (Table 2) and gently air-pumped for 5 days under steady ambient temperature, RH and CO_2_ levels. Post adaptation to hydroponic full Hoagland media, plants were taken out of their beakers (exc. Control), washed thoroughly with DDW and transferred to a premixed single-microelement-deficient Hoagland solution. Plants were visually examined daily for signs of nutrient deficiency. Once identified, each deficient plant was treated for its corresponding deficiency, by 20–30 µl of either common nutrient formulations or liposome-entrapped formulation in the commonly used concentration of 0.2%, applied every 48 hours.

**Table 2:** Hoagland solution formulation modified from Epstein, E.^39^.

Component	0.5 Hoagland solution (optimum) [mg/l]	Stock ×10 [g/l]	Stock ×1000 [g/l]
**Macroelements**			
Ca(NO_3_)_2_ **·** 4H_2_0	720	7.2	
KNO_3_	460.98	4.61	
MgSO_4_ **·** 7H_2_O	493	4.93	
KH_2_PO_4_	272	2.72	
Iron (Sprint 138 iron chelate)	100	1.0	
**Microelements**			
H_3_BO_3_	2.86		2.09
MnCl_2_ **·** H_2_O	1.8		1.8
ZnSO_4_ **·** 7H_2_O	0.22		0.22
CuSO_4_	0.08		0.08
NaMoO_4_ **·** 2H_2_O	0.017		0.017

Deficient formulas were obtained by depriving the solution from either of the single-added minerals, focusing on minerals which essentially present a distinctively observable effect over the plant.

We used Hoagland setup^38^ and Hoagland-based solution modified by Epstein, E.^39^, along with single-genus “Ikram” Tomato seeds, in order to knowingly address an iron (Fe) deficiency. Plants were given 5 days to adjust to full Hoagland media prior to introduction of Fe-deficient Hoagland media. Upon emergence of initial deficiency signals as well as decelerated new growth, plants were treated by both liposomal-encapsulated and free-form Fe-based compounds, implemented over single apical leaflet one leaf below new growth.

## Electronic supplementary material

Supplementary Information
