# Supplementary material for: Therapeutic nanoparticles penetrate leaves and deliver nutrients to agricultural crops
Source: Sci Rep. 2018 May 17;8:7589. doi: 10.1038/s41598-018-25197-y (PMC5958142; doi:10.1038/s41598-018-25197-y)
Supplement: Supplementary file 1 — Supplementary Information [file 41598_2018_25197_MOESM1_ESM.pdf]

Therapeutic nanoparticles penetrate leaves and deliver nutrients to agricultural crops

Avishai Karny,[1] Assaf Zinger,[1] Ashima Kajal,[1] Janna Shainsky-Roitman[1] and Avi Schroeder[1,*]

[1]Department of Chemical Engineering, Technion – Israel Institute of Technology, Haifa 32000, Israel

SUPPLEMENTARY INFORMATION

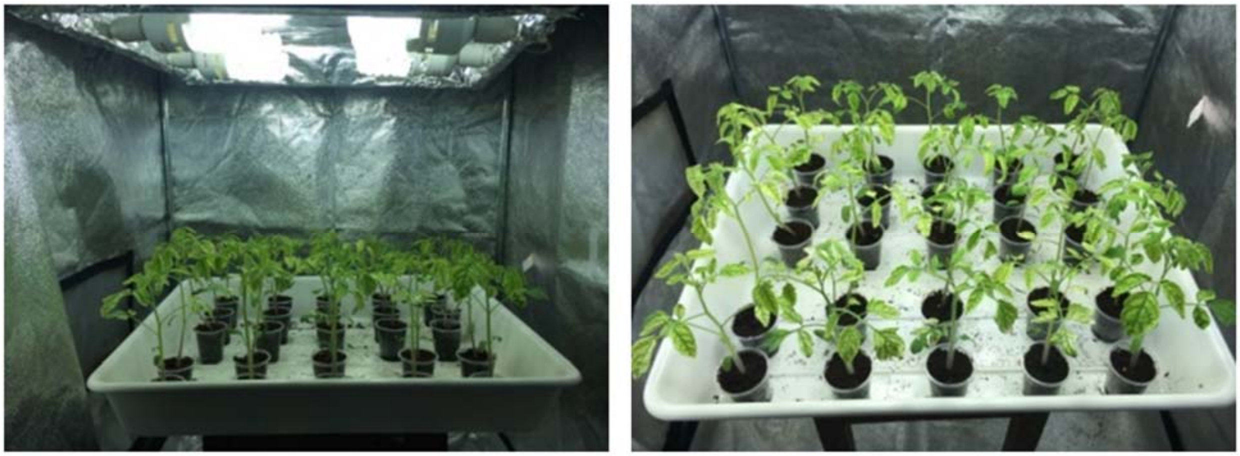

Supplementary 1. Tomato plants grown in micronutrient-poor growth substrate prior to treatment. At 4 weeks of age (5 fully grown leaves) plants show acute deficiencies characterized by severe epinasty and chlorosis in between leaf veins, symptoms attributed to Magnesium and Iron deficiencies.

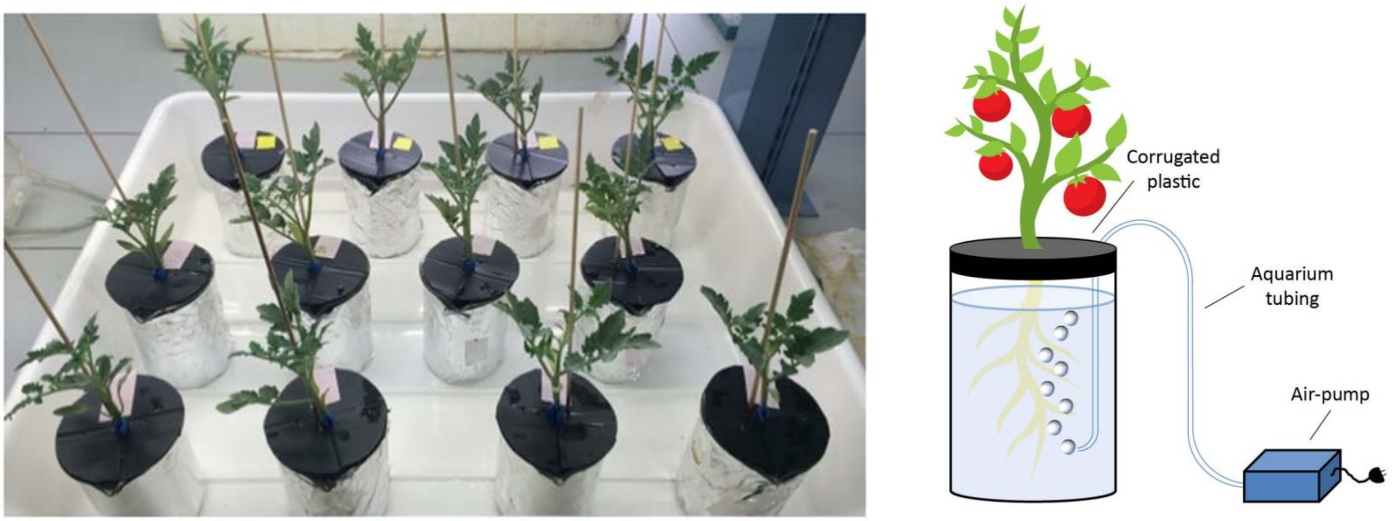

Supplementary 2. Hydroponic system setup. (left) Tomato plant seedlings were grown in nursery and transferred to 250 ml beakers containing full Hoagland solution for a 5 day adjustment to hydroponic media, later to be changed to Fe-deficient Hoagland solution. (right) Each plant setup included continuous adequate air pumping throughout the experiment.
